# The antimalarial drug mefloquine enhances *TP53* premature termination codon readthrough by aminoglycoside G418

**DOI:** 10.1371/journal.pone.0216423

**Published:** 2019-05-23

**Authors:** Michael W. Ferguson, Chloe A. N. Gerak, Christalle C. T. Chow, Ettore J. Rastelli, Kyle E. Elmore, Florian Stahl, Sara Hosseini-Farahabadi, Alireza Baradaran-Heravi, Don M. Coltart, Michel Roberge

**Affiliations:** 1 Department of Biochemistry and Molecular Biology, University of British Columbia, Vancouver, British Columbia, Canada; 2 Department of Chemistry, University of Houston, Houston, Texas, United States of America; Roswell Park Cancer Institute, UNITED STATES

## Abstract

Nonsense mutations constitute ~10% of *TP53* mutations in cancer. They introduce a premature termination codon that gives rise to truncated p53 protein with impaired function. The aminoglycoside G418 can induce *TP53* premature termination codon readthrough and thus increase cellular levels of full-length protein. Small molecule phthalimide derivatives that can enhance the readthrough activity of G418 have also been described. To determine whether readthrough enhancers exist among drugs that are already approved for use in humans, we tested seven antimalarial drugs for readthrough of the common R213X *TP53* nonsense mutation in HDQ-P1 breast cancer cells. Mefloquine induced no *TP53* readthrough activity as a single agent but it strongly potentiated readthrough by G418. The two enantiomers composing pharmaceutical mefloquine potentiated readthrough to similar levels in HDQ-P1 cells and also in SW900, NCI-H1688 and HCC1937 cancer cells with different *TP53* nonsense mutations. Exposure to G418 and mefloquine increased p53 phosphorylation at Ser15 and *P21* transcript levels following DNA damage, indicating p53 produced via readthrough was functional. Mefloquine does not appear to enhance readthrough via lysosomotropic effects as it did not significantly affect lysosomal pH, the cellular levels of G418 or its distribution in organellar or cytosolic fractions. The availability of a readthrough enhancer that is already approved for use in humans should facilitate study of the therapeutic potential of *TP53* readthrough in preclinical cancer models.

## Introduction

Nonsense mutations are single base substitutions that inactivate genes by introducing a premature termination codon (PTC). The presence of a PTC in mRNA causes the synthesis of truncated protein and can trigger degradation of the mRNA via nonsense-mediated decay. Nonsense mutations constitute ~10% of the cancer-associated mutations found in the tumor suppressor gene *TP53* and rescue of nonsense mutations in tumour suppressor genes has been proposed as a cancer therapy strategy [1–4].

Nonsense mutations can be rescued by PTC readthrough, a process that enables synthesis of full-length protein from mRNAs harboring nonsense mutations. Aminoglycoside antibiotics were the first chemical agents shown to induce PTC readthrough in eukaryotes [5,6]. Their binding to the decoding center of cytosolic ribosomes provokes a structural change that enables pairing of a near-cognate aminoacyl-tRNA to the PTC and thus incorporation of an amino acid residue instead of translation termination [7,8]. Unlike PTCs, termination codons are resistant to readthrough because they are in proximity to 3′ untranslated regions sequences and the poly(A) tail which strongly promote rapid and efficient translation termination [9]. Gentamicin, the most extensively tested readthrough aminoglycoside, elicits significant readthrough only at mg/ml concentrations in cell culture [10], much higher than the ~10 μg/ml blood levels above which gentamicin can cause nephrotoxicity and ototoxicity.

Recent efforts have focused on identifying more potent readthrough aminoglycosides and on optimizing their structure to reduce their toxicity. G418, NB54, NB84 and NB124 show improved readthrough potency in cellular assays but they still lack the low- to sub-μM activity desirable for drug candidates [10–12]. It has also been observed that the PTC readthrough activity of aminoglycosides can be increased by other compounds. Inhibitors of nonsense-mediated decay can modestly enhance PTC readthrough by aminoglycosides [13]. A cell-based screen also identified a family of phthalimide derivatives with unknown mechanism of action that are capable of strongly enhancing readthrough by the aminoglycoside G418 [14]. Combination treatment may enable PTC readthrough at lower, less toxic aminoglycoside doses. Developing a combination therapy using two experimental drugs is extremely challenging from scientific, economic and regulatory perspectives. However, combination therapies are considered easier to develop when one of the components is already approved as a single agent [15]. This consideration led us to search for PTC readthrough enhancers among drugs that are already approved for use in humans. Here we report that the antimalarial drug mefloquine can considerably enhance readthrough by G418 in cancer cells harboring different *TP53* nonsense alleles.

## Materials and methods

### Cancer cell lines

HDQ-P1 cells were purchased from the German Collection of Microorganisms and Cell Cultures, and HCT116, SW900, NCI-H1688, HCC1937 and NCI-H1299 cells from American Type Culture Collection. NCI-H1299 cells stably expressing p53 R213X (TGA) were generated in our laboratory. HCT116 cells are homozygous for wild type *TP53* and the following cancer cell lines harbor homozygous nonsense mutations in the *TP53* gene: HDQ-P1 (R213X), SW900 (Q167X), NCI-H1688 (Q192X), HCC1937 (R306X) [14]. HDQ-P1 and HCT116 cells were cultured in high glucose Dulbecco’s modified Eagle medium (DMEM, Sigma-Aldrich) supplemented with 10% (vol/vol) fetal bovine serum (FBS, Sigma-Aldrich) and 1× antibiotic–antimycotic (Gibco/Thermo Fisher Scientific) at 37°C and 5% (vol/vol) CO_2_. SW900, NCI-H1688, HCC1937, and NCI-H1299 cells were cultured in RPMI-1640 medium (Sigma-Aldrich) supplemented with 10% (vol/vol) FBS (Sigma-Aldrich) and 1× antibiotic–antimycotic (Gibco/Thermo Fisher Scientific) at 37°C and 5% (vol/vol) CO_2_.

### Chemicals

**Fig 1:**
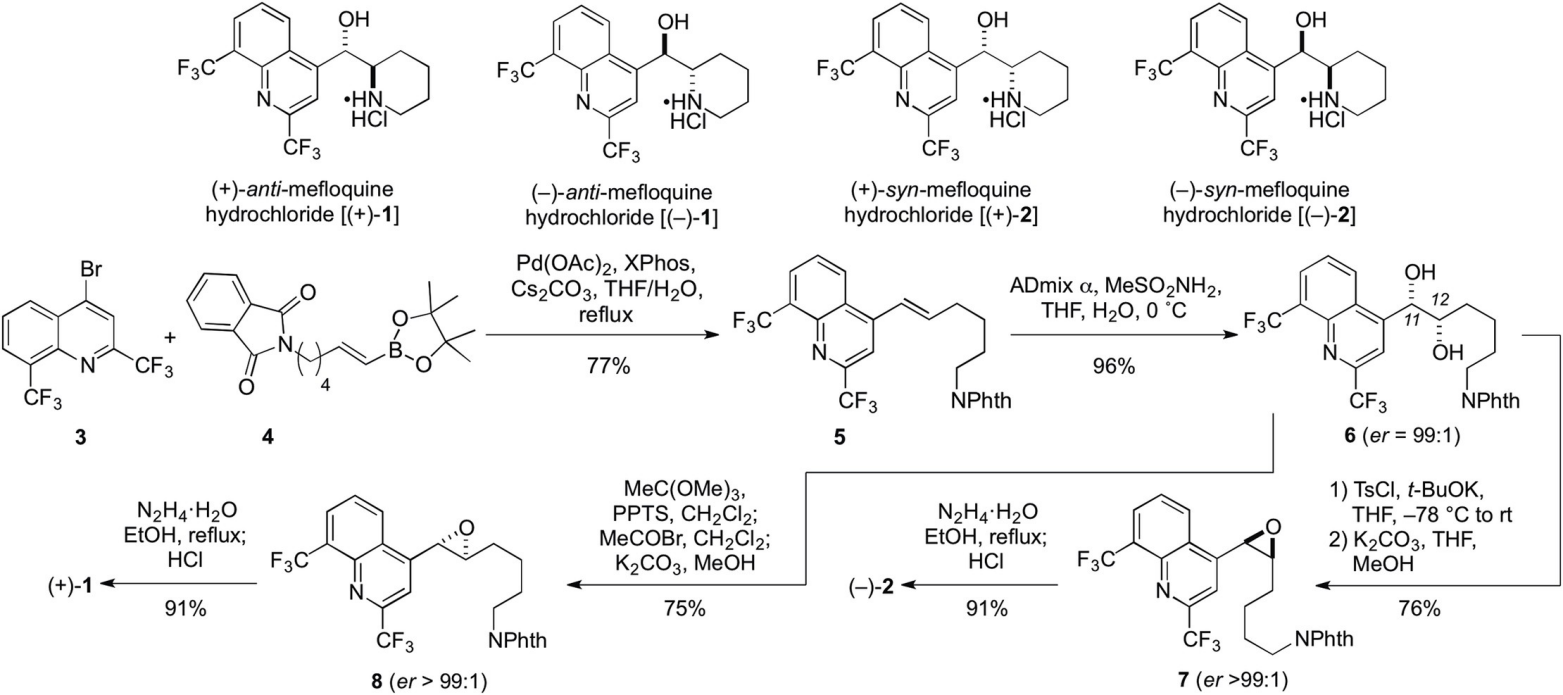
Asymmetric total synthesis of (+)-*Anti*-mefloquine [(+)-1] and (–)-*anti*-mefloquine [(–)-2].

Chloroquine diphosphate (C6628), hydroxychloroquine sulfate (90527), amodiaquine dihydrochloride dihydrate (A2799) and primaquine bisphosphate (160393) were purchased from Sigma-Aldrich and solubilized in distilled water. Quinine (22620), mefloquine hydrochloride (M2319) and pamaquine naphthoate (S509515) were purchased from Sigma-Aldrich and solubilized in dimethyl sulfoxide. G418 was purchased from MicroCombiChem. Mefloquine stereoisomers (+)-*anti*-mefloquine hydrochloride [(+)-**1**] and (-)-*syn*-mefloquine hydrochloride [(-)-**2**] were synthesized and purified as described [16,17]. Mefloquine stereoisomers (-)-*anti*-mefloquine hydrochloride [(-)-**1**] and (+)-*syn*-mefloquine hydrochloride [(+)-**2**] were synthesized in an analogous manner (Fig 1). Briefly, compound **5** was prepared via coupling of **3** and **4**. Using known synthetic procedures, **5** was converted to diol **6** by treatment with ADmix α, which was then converted to (+)-**1** using a three-step sequence. (–)-**2** was also generated from **6** in three steps, but using a different synthetic sequence. (–)-**1** and (+)-**2**, the enantiomeric forms of (+)-**1** and (–)-**2**, respectively, were prepared in an analogous way, but using Admix β.

### Automated p53 immunofluorescence assay

HDQ-P1 cells were seeded into PerkinElmer View 96-well plates at 11,000 cells per well. The next day, cells were treated with compounds for 48 h. Cells were then fixed, stained, and immunolabelled with DO-1 mouse α-p53 monoclonal primary antibody (Santa Cruz) and Alexa 488-conjugated goat α-mouse secondary antibody (Thermo Fisher Scientific) as previously described [14]. Plates were then covered with a black membrane and stored at 4°C until analyzed. Imaging was performed using a Cellomics ArrayScan VTI automated fluorescence microscope and 15 fields per well at 20x magnification were captured. Results were expressed as the average nuclear p53 immunofluorescence intensity [14].

### Automated capillary electrophoresis western analysis

p53 levels were measured as previously described [14]. Results were analyzed by built-in Compass software (ProteinSimple) and expressed both qualitatively as pseudo-blots, and quantitively by FL:FL ratio (the ratio of full-length p53 to full-length p53 in cells exposed to G418 alone). Pseudo-blots illustrate chemiluminescent peak intensities of protein targets as determined by Compass software. FL:FL ratios were calculated by normalizing p53 and vinculin peak intensities to the peak intensity of vinculin in untreated cells to account for variations in protein loading. Relative changes in FL-p53 expression were then calculated by comparing FL-p53 chemiluminescence of samples to cells treated with G418 alone. Phosphorylated p53 was measured using Calbiochem PC386 antibody (1:50), TPP1 with Abcam ab54685 α-TPP1 antibody (0.8 μg/ml), and GAPDH with Abcam ab9485 α-GAPDH antibody (1:400).

### Detection of *TP53* and *P21* mRNA expression by RT-qPCR

H1299, HDQ-P1, and HCT116 cells were seeded at 150,000 cells per well in 6-well plates and treated the following day with compounds. Cells were exposed to 4 Gy ionizing radiation 48 h after addition of compound and 24 h later, the medium was removed by aspiration, and RNA was extracted using the RNeasy Plus Mini Kit (Qiagen). cDNA synthesis was then performed using the High-Capacity RNA-to-cDNA Kit (Thermo Fisher Scientific). The ABI StepOnePlus Real-Time PCR system was employed to detect changes in *TP53* and *P21* mRNA levels using primers described previously [14]. *GAPDH* served as the reference gene. Relative changes in mRNA levels were determined using the ΔΔCt method.

### Measurement of intracellular G418

HDQ-P1 cells were seeded at 1,300,000 cells per 15-cm diameter tissue culture plate. Following drug treatment, cell monolayers were rinsed 5x with 3 ml ice cold PBS and then trypsinized for 10 min at 37°C. The suspended cells were diluted in ice cold PBS, counted, and 340,000 cells were pelleted by centrifugation at 300 g for 2 min at 4°C. Cell pellets were washed one additional time in ice cold PBS and intracellular G418 concentrations were measured as follows. For total cell G418 levels, pellets were suspended in NP-40 lysis buffer (150 mM NaCl, 50 mM Tris-HCl (pH 7.5), 1% NP-40) and incubated on ice for 30 min. The samples were centrifuged at 20,000 g for 10 min at 4°C, and the supernatants (total cell) were collected. In parallel, samples were subjected to a crude subcellular fractionation to separate cytosolic and organellar fractions as previously described, with slight modifications [18]. After rinsing, trypsinizing and washing cell pellets in ice cold PBS, cells were exposed to digitonin lysis buffer (100 μg/ml digitonin, 150 mM NaCl, 50 mM Tris-HCl (pH 7.5)) and incubated on ice for 10 min. The samples were centrifuged at 20,000 g for 10 min at 4°C, and the supernatants (cytosolic fraction) were collected. The remaining pellets were resuspended in NP-40 lysis buffer and incubated on ice for 30 min. The samples were centrifuged at 20,000 g for 10 min at 4°C, and the supernatants (organellar fraction) were collected. G418 levels were determined using an indirect competitive gentamicin ELISA kit (Creative Diagnostics, DEIA047), according to the manufacturer’s protocol, except that standard curves were generated using G418 diluted in NP-40 lysis buffer or in digitonin lysis buffer.

### Statistics

Data are presented as mean ± SD of the number of biological replicates indicated in the figure legends.

## Results

Several approved antimalarial drugs are related to quinine [19]. They are hydrophobic weak bases that can diffuse across biological membranes and accumulate inside acidic intracellular structures such as lysosomes [20]. Given the observation that aminoglycosides tend to localize intracellularly to lysosomes [21], we speculated that quinine analogs might affect PTC readthrough by aminoglycosides.

**Fig 2:**
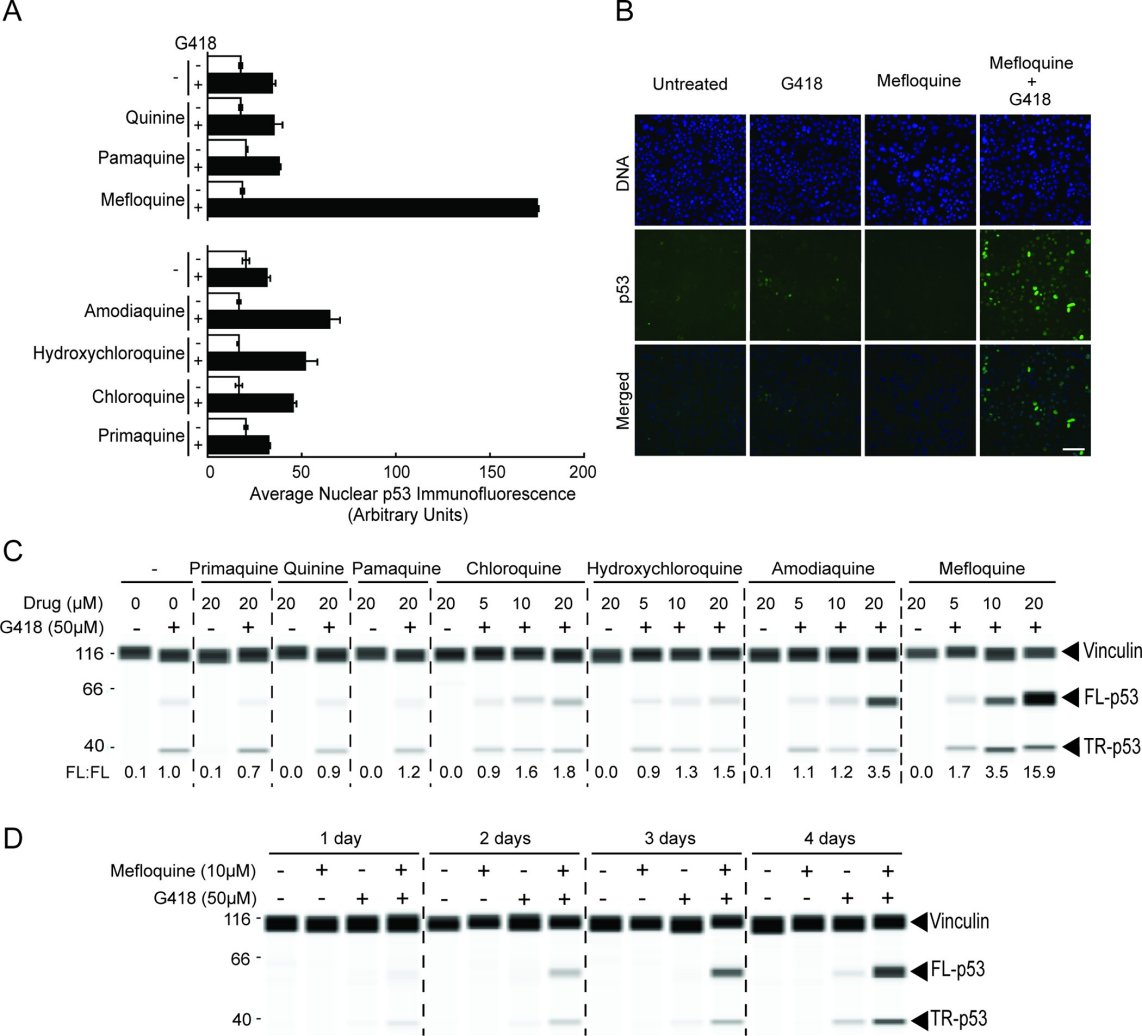
Identification of mefloquine as a potent enhancer of G418-induced *TP53* PTC readthrough. (A) Automated fluorescence microscopy analysis of PTC readthrough. HDQ-P1 cells in 96-well plates were treated for 48 h with 20 μM of the indicated drugs in the presence or absence of 50 μM G418 and nuclear p53 levels were measured. Data are expressed as the mean ± SD (n = 6 biological replicates for controls, n = 3 biological replicates for drug treatments) and were collected from two independent experiments, represented by the break in the graph. (B) Representative images of p53 immunofluorescence from A. Blue shows DNA staining and green is p53 immunofluorescence (scale bar, 100 μm). (C) Western analysis of p53 expression following 48 h treatment. Samples were analysed by automated quantitative capillary electrophoresis western analysis with a p53 antibody to measure full-length p53 (FL-p53) and truncated p53 (TR-p53), and a vinculin antibody. The chemiluminescence signals are shown as pseudo-blots. The amount of FL-p53 was normalized to that of vinculin. FL:FL represent the ratio of FL-p53 in treated samples to FL-p53 in cells exposed to G418 alone. (D) Western analysis of p53 expression over time. Samples were analysed by automated quantitative capillary electrophoresis western analysis as above.

We examined the effect of seven approved antimalarial drugs on PTC readthrough by the aminoglycoside G418 using the HDQ-P1 *TP53* readthrough assay [10,14]. HDQ-P1 human breast carcinoma cells carry a homozygous *TP53* R213X nonsense mutation that is susceptible to readthrough by aminoglycosides such as G418 and NB124 [10,12]. HDQ-P1 cells express little to no detectable p53 protein and PTC readthrough enables the production of p53, which translocates to the nucleus [14]. Readthrough was first tested using a high-throughput 96-well plate quantitative automated immunofluorescence microscopy assay that measures nuclear p53 [14]. Cells seeded in 96-well plates were exposed for 48 h to 20 μM quinine, pamaquine, mefloquine, amodiaquine, hydroxychloroquine, chloroquine or primaquine without or with 50 μM G418, a submaximal concentration for PTC readthrough. Nuclear p53 was then determined in 500 cells per well in triplicate wells. Untreated cells showed marginal levels of p53 immunofluorescence and treatment with 50 μM G418 produced a modest increase in p53 (Fig 2A and 2B), representing low-level PTC readthrough. As single agents, none of the seven drugs elicited any detectable increase in nuclear p53 immunofluorescence (Fig 2A), indicating that they do not induce PTC readthrough. By contrast, combining 50 μM G418 with 20 μM chloroquine, hydroxychloroquine, amodiaquine or mefloquine increased nuclear p53 more than G418 alone, with mefloquine eliciting the highest signal (Fig 2A and 2B).

To formally demonstrate PTC readthrough, the cells were subjected to automated capillary electrophoresis western analysis to quantitate full-length p53, the readthrough product. The amount of full-length p53 was normalized to vinculin, used as a protein loading control, and expressed relative to the amount of full-length p53 in cells exposed to G418 alone. Untreated cells showed essentially no full-length p53 while exposure to 50 μM G418 induced some readthrough, as indicated by the appearance of full-length p53 (Fig 2C). G418 also increased the levels of truncated p53, as previously described [14]. Here again, none of the seven drugs induced p53 readthrough when used alone (Fig 2C). In combination with G418, chloroquine and hydroxychloroquine caused a <2-fold increase in readthrough while amodiaquine increased readthrough 3.5-fold, and mefloquine enhanced readthrough 15.9-fold (Fig 2C), consistent with the immunofluorescence data. In a time-course experiment, readthrough by mefloquine and G418 was detectable within 24 h of exposure and this effect increased during 4 days of treatment (Fig 2D). Thus, mefloquine was identified as a strong enhancer of PTC readthrough.

**Fig 3:**
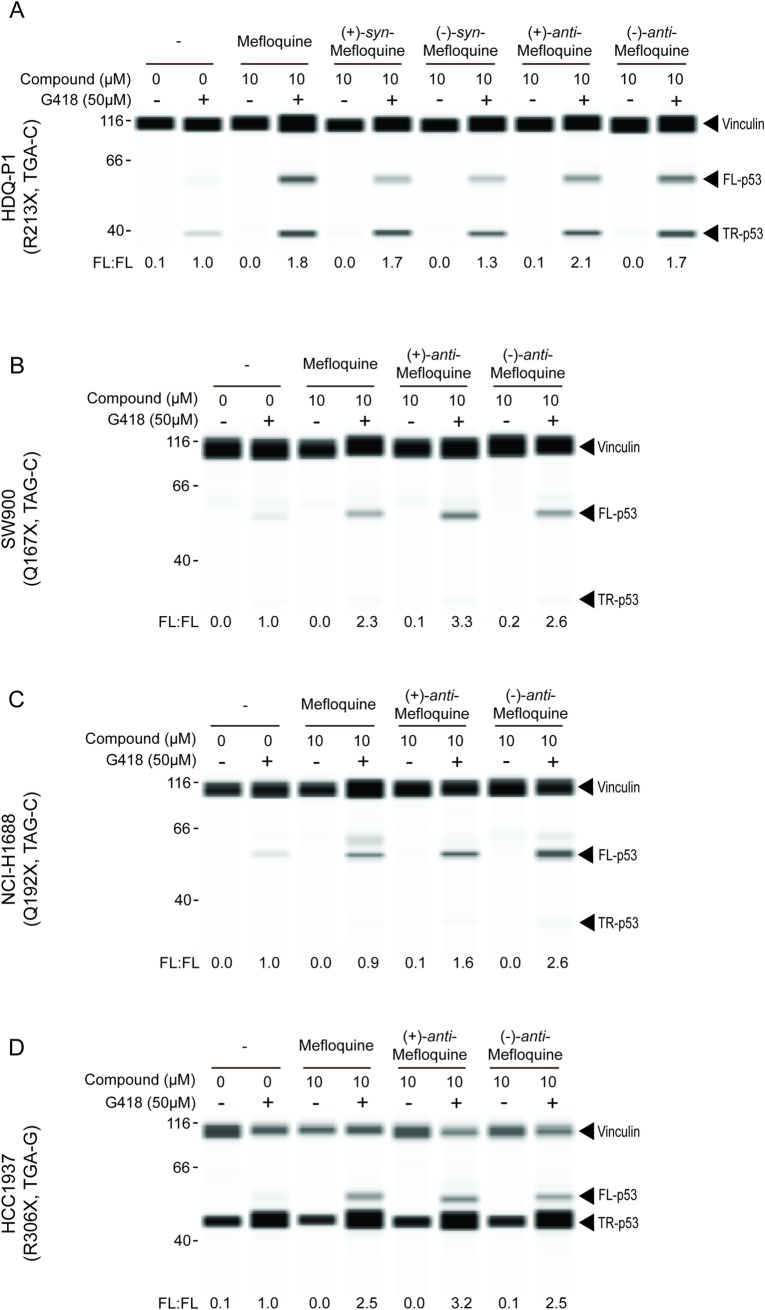
**PTC readthrough enhancement by mefloquine and individual stereoisomers in HDQ-P1 cells after 48 h exposure (A) and in human cancer cell lines harboring different nonsense mutations in *TP53* after 72 h exposure (B-D).** FL-p53 was measured and expressed as in Fig 1.

Mefloquine is a chiral molecule that contains two asymmetric carbon centers and thus encompasses four stereoisomers (Fig 1). The pharmaceutical product is composed of a racemic mixture of the two *anti*-enantiomers [(+)-**1** and (–)-**1**]. To investigate the contributions of individual mefloquine stereoisomers to readthrough enhancement, the four stereoisomers were synthesized and their activity was compared to that of pharmaceutical mefloquine. (+)-*Anti*-mefloquine and (–)-*anti*-mefloquine [(+)-**1** and (–)-**1**, respectively], the single enantiomers of the racemic pharmaceutical form of mefloquine, enhanced readthrough to similar levels (Fig 3A). The non-pharmaceutical (+)-*syn*-mefloquine and (–)-*syn*-mefloquine [(+)-**2** and (–)-**2**, respectively] also enhanced readthrough, but the latter appeared slightly less active (Fig 3A).

The propensity of PTCs to undergo readthrough is influenced by a number of factors including the sequence of the termination codon, with TGA and TAG showing higher basal and drug-induced readthrough than TAA, the bases adjacent to the PTC, the position of the PTC within the mRNA sequence, as well as extragenic determinants [22,23]. We next investigated whether mefloquine potentiates PTC readthrough by G418 in cancer cell lines with nonsense mutations at different positions in the *TP53* gene. SW900, NCI-H1688, and HCC1937 cell lines with homozygous Q167X, Q192X, or R306X nonsense mutations were exposed to combinations of 10 μM mefloquine and 50 μM G418 for 72 h and full-length p53 levels were analyzed. Mefloquine and its individual *anti*-enantiomers enhanced PTC readthrough in all cell lines (Fig 3B–3D), and to roughly equivalent levels. This result shows mefloquine can enhance *TP53* readthrough at positions other than R213X and in cancer cell lines with different genetic backgrounds.

We next examined whether the level of full-length p53 produced by G418 and mefloquine was sufficient to increase p53 function. Wild type p53 is activated in response to cellular stresses such as DNA damage and a key early regulatory step in the activation process is phosphorylation of p53 at serine 15 [24,25]. Three human cancer cell lines–HCT116 (WT *TP53*), HDQ-P1 (*TP53*-R213X) and H1299-p53 R213X (*TP53*-null H1299 cells stably expressing *TP53*-R213X cDNA) were exposed to 10 μM mefloquine in the absence or presence of 50 or 100 μM G418. After 24 h, the cells were exposed or not to ionizing radiation to induce DNA damage and they were harvested after an additional 24 h. The levels of p53 and phosphorylated p53 were then measured by automated capillary western analysis.

**Fig 4:**
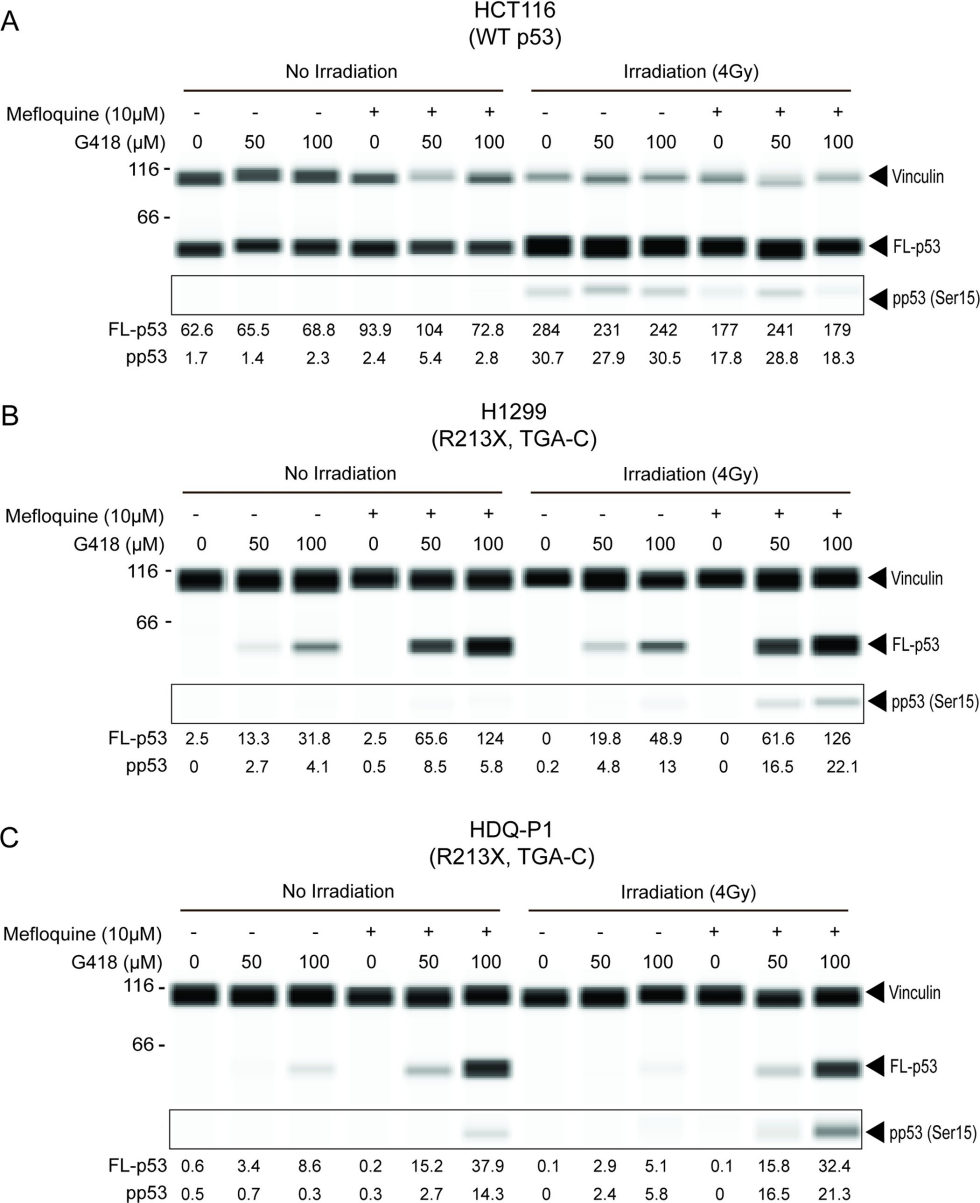
Effect of ionizing radiation on the Ser15 phosphorylation of p53 produced by PTC readthrough. The indicated cancer cells were treated with compounds for 48 h and exposed to 4 Gy ionizing radiation 24 h prior to harvesting. Following treatment, p53 and phosphorylated p53 (pp53 Ser15) levels were measured by automated capillary electrophoresis western analysis. Vinculin and FL-53 were measured in the same capillary while pp53 (Ser15) was measured in a duplicate capillary, indicated by the box. Numbers for FL-p53 and pp53 are chemiluminescence values normalized to that of vinculin and divided by 1000.

Without irradiation, HCT116 cells, used as a WT control, contained readily measurable p53 levels but barely detectable phosphorylated p53. Irradiation increased both the level of p53 and its phosphorylation (Fig 4A). The increased p53 level is consistent with Ser15 phosphorylation disrupting the interaction of p53 with MDM2 and reducing p53 degradation [26]. Exposure to G418 and mefloquine, alone or in combination, did not increase p53 levels or phosphorylation in non-irradiated HCT116 cells and it did not significantly affect p53 levels or phosphorylation in irradiated samples (Fig 4A), indicating that these compounds do not affect pathways that control p53 expression or Ser15 phosphorylation.

H1299 cells overexpressing *TP53*-R213X and HDQ-P1 cells with endogenous *TP53*-R213X had barely detectable levels of full-length p53 or Ser15 phosphorylation in the absence of drug treatment, without or with irradiation (Fig 4B and 4C). Both cell lines expressed full-length p53 upon exposure to G418 and mefloquine, and irradiation increased Ser15 phosphorylation (Fig 4B and 4C). The truncated p53 band showed no Ser15 phosphorylation under any condition (not shown).

**Fig 5:**
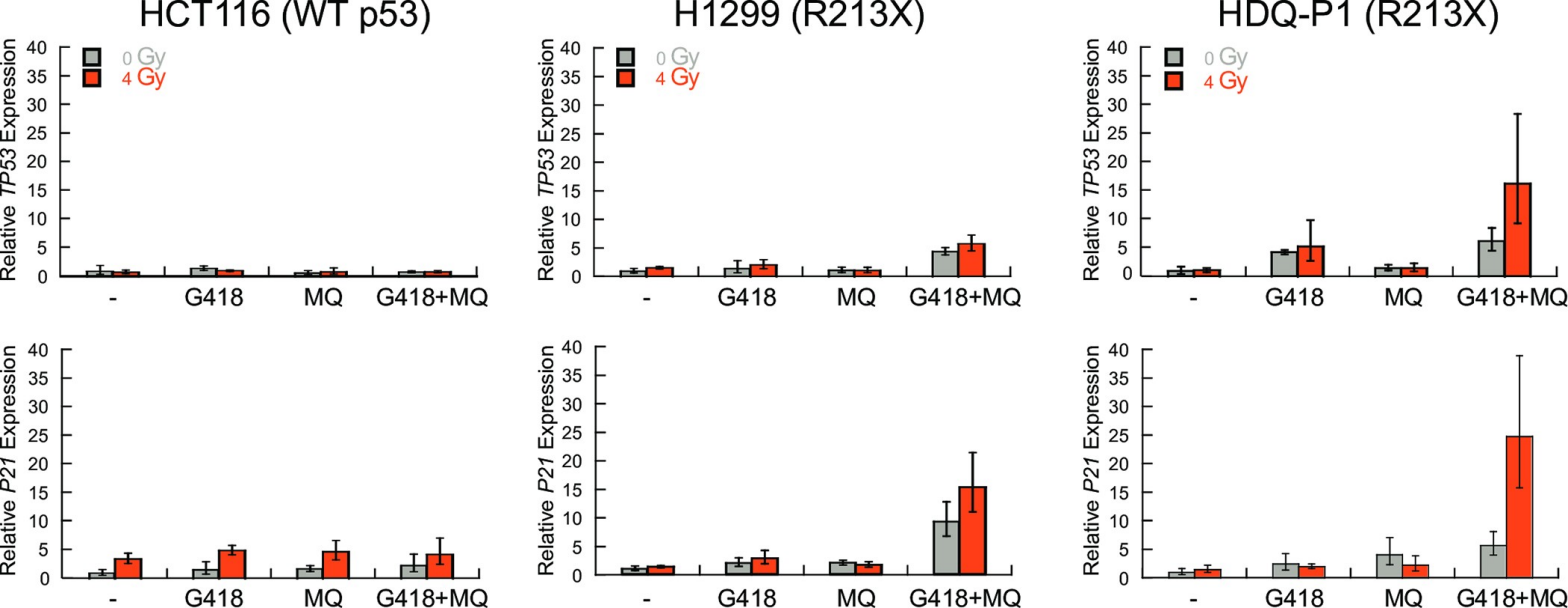
Effect of mefloquine, G418 and irradiation on *TP53* and *P21* expression. Cells were treated as indicated and relative *TP53* and *P21* transcript levels were quantified by RT-qPCR. Data are expressed as the mean ± SD (n = 3 biological replicates). *GAPDH* served as the reference gene.

Phosphorylated WT p53 can transcriptionally activate downstream targets such as *P21* [24,25]. We next measured changes in both *TP53* and *P21* transcript levels under readthrough-inducing conditions. The cells were exposed to 10 μM mefloquine and 100 μM G418 for 72 h and exposed or not to 4 Gy of ionizing radiation 48 h after compound addition. Exposure of control HCT116 cells (WT *TP53*) to G418 alone, mefloquine alone, or G418 and mefloquine, without or with ionizing radiation did not affect *TP53* transcript levels (Fig 5), consistent with p53 being regulated post-translationally [26]. However, *P21* transcript levels increased significantly following irradiation (Fig 5).

Exposure of H1299-p53 R213X cells to G418 alone or mefloquine alone did not significantly increase *TP53* transcript levels but exposure to the combination of G418 and mefloquine did (Fig 5). This increase in transcript level is likely a consequence of PTC readthrough, which helps transcripts bearing PTCs escape degradation by nonsense-mediated decay [14]. Exposure of H1299-p53 R213X cells to G418 and mefloquine caused a large increase in *P21* expression, which was further enhanced by irradiation (Fig 5). In HDQ-P1 cells, exposure to G418 alone or G418 and mefloquine increased *TP53* transcript levels and irradiation of cells exposed to G418 and mefloquine resulted in greatly increased expression of the *P21* transcript (Fig 5), correlating with the high p53 Ser15 phosphorylation observed in Fig 4. Taken together, these results indicate that readthrough enhancement by mefloquine produces functional p53 protein capable of upregulating the expression of its downstream target *P21*.

**Fig 6:**
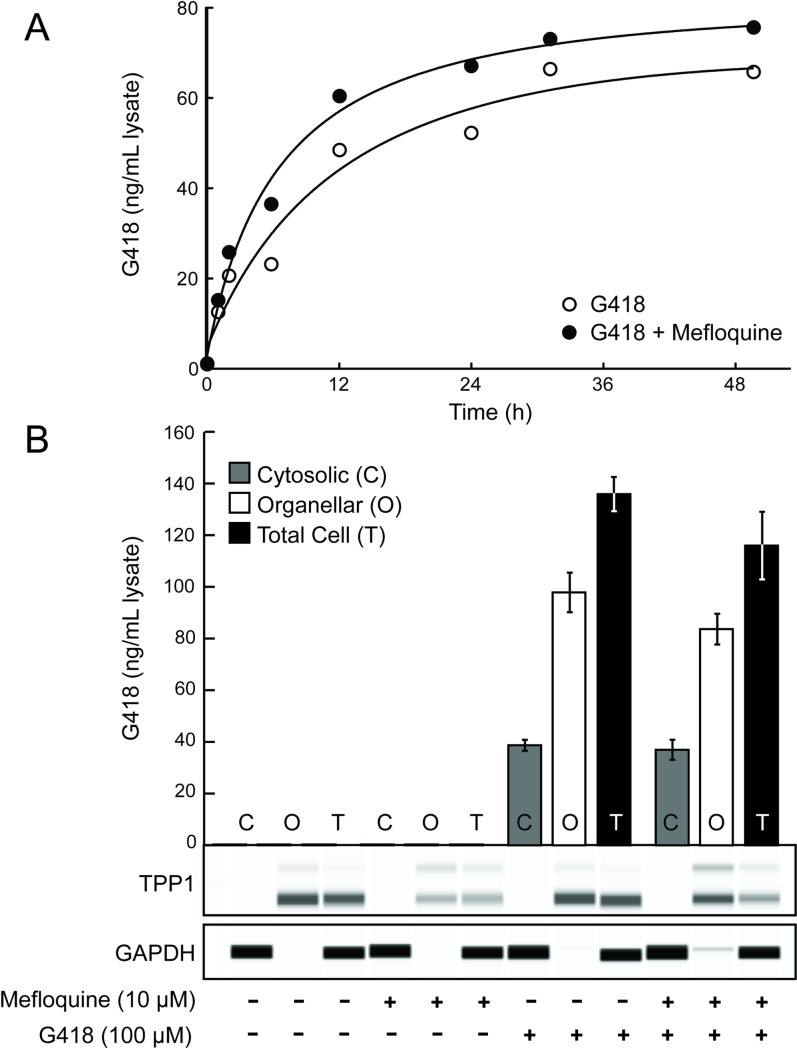
G418 accumulation in cells. (A) G418 was added with or without mefloquine at t = 0 and its total intracellular levels were measured at the indicated times. The solid line shows logarithmic curve fit. (B) Cells were exposed or not to G418 and mefloquine for 24 h and subjected to crude subcellular fractionation to determine cytosolic and organellar G418 concentrations, as well as total cellular G418 concentration. Western analysis shows the amount of TPP1, a lysosomal protein, and GAPDH, a cytosolic protein, in each fraction. Data are expressed as the mean ± SD (n = 3 biological replicates).

To address the mechanism by which mefloquine enhances PTC readthrough, we first investigated whether it increases the cellular levels of G418. HDQ-P1 cells were exposed to 100 μM G418 without or with 10 μM mefloquine for different times. They were rinsed rapidly and extensively to eliminate extracellular G418, and lysed in buffer containing NP-40 to solubilize plasma and intracellular membranes. G418 was measured in lysates using a commercial gentamicin ELISA kit and normalized on a cell number basis. G418 accumulation in cells followed a logarithmic growth, reaching half-maximal value at ~6 h and a plateau at ~48 h (Fig 6A). Mefloquine slightly increased the rate and levels of intracellular G418. However, in a separate experiment, the cellular levels of G418 were slightly lower after 24 h exposure to mefloquine (Fig 6B), indicating that mefloquine does not significantly affect G418 cellular accumulation.

In kidney cells, gentamicin segregates largely to lysosomes [21,27]. We next asked whether mefloquine might enhance PTC readthrough by causing the release of G418 from lysosomes, thereby increasing its cytosolic concentration and binding to ribosomes. Cells were exposed to digitonin to selectively permeabilize the plasma membrane, but not intracellular membranes, and then centrifuged to separate cytosolic components from organellar components [18]. The organellar fraction was subsequently solubilized with NP-40 to release its contents. To optimize the assay conditions, HDQ-P1 cells were first incubated with different concentrations of digitonin and the release of the cytosolic protein GAPDH and the lysosomal lumen protein TPP1 was monitored by western analysis. A digitonin concentration of 100 μg/ml was optimal as it released most cellular GAPDH but little to no TPP1 (S1 Fig).

Next, HDQ-P1 cells were exposed to G418 without or with mefloquine for 24 h and the cytosolic and organellar fractions were separated. Exposure of cells to mefloquine or G418 alone did not alter the distribution of GAPDH and TPP1 compared to untreated cells (Fig 6B), indicating that these compounds did not compromise the integrity of cellular membranes. G418 was predominantly in the organellar fraction in HDQ-P1 cells and remained predominantly in the organellar fraction after mefloquine treatment (Fig 6B). Therefore, mefloquine did not enhance PTC readthrough by increasing the concentration of G418 in the cytosol. Additionally, at concentrations that enhance PTC readthrough, mefloquine did not reduce lysotracker red staining in HDQ-P1 cells (S2 Fig), indicating that its intracellular levels were not sufficiently high to reduce lysosomal acidification.

## Discussion

This study shows that *TP53* PTC readthrough by the aminoglycoside G418 can be strongly enhanced by mefloquine. The related aminoglycoside gentamicin is known to accumulate in lysosomes after entering kidney proximal tubule cells [21] and we observe that G418 also localizes predominantly to the organellar fraction of HDQ-P1 breast cancer cells. The measurements of intracellular G418 reported here do not support the hypothesis that mefloquine increases readthrough by raising the intracellular levels of G418 or by causing the redistribution of G418 from lysosomes to the cytosol.

Accumulation of weak bases such as quinine derivatives in lysosomes can neutralize their acidic pH and inhibit the activity of lysosomal hydrolases, leading to a number of downstream effects that include inhibition of autophagic flux, increased oxidative stress and p53 activation [28,29]. It is therefore possible that enhancement of PTC readthrough is a consequence of impairment of lysosomal function. However, our observation that mefloquine enhanced readthrough at concentrations that did not reduce lysotracker red staining in HDQ-P1 cells indicate that its intracellular levels were not sufficiently high to reduce lysosomal acidification and impair lysosomal function and argues against this mechanism.

An alternative possibility is that mefloquine might directly target the translation machinery. Interestingly, mefloquine has been shown to bind to the 80S ribosome of the malaria parasite *P*. *falciparum* [30] and to partially inhibit protein translation. The primary mefloquine binding site is in the GTPase-associated centre of the ribosome, where the (+) enantiomer binds to ribosomal protein uL13 [30]. The mefloquine-binding pocket of uL13 is incompletely conserved between *P*. *falciparum* and human, the latter showing two non-conservative substitutions that may reduce binding affinity to human cytosolic ribosomes [30]. Whether mefloquine binds to human ribosomes at concentrations that potentiate PTC readthrough remains to be investigated. Nonetheless, it is conceivable that concurrent binding of mefloquine and G418 to a ribosome might induce a conformation that is more conducive to readthrough than binding of G418 alone. On the other hand, our observation that the (-) enantiomer of mefloquine can enhance PTC readthrough is not consistent with this hypothesis as it does not appear to bind to the GTPase-associated centre of *P*. *falciparum* ribosomes, although it interacts with a secondary site [30].

PTC readthrough has been proposed as a therapeutic strategy to reestablish p53 function in cancer patients whose tumours bear *TP53* nonsense mutations [1,2]. Our observation that irradiation of HDQ-P1 breast cancer cells exposed to G418 and mefloquine increases p53 Ser15 phosphorylation and *P21* expression indicates that p53 function is at least partially restored. Unfortunately, the PTC readthrough aminoglycosides available to date are not very potent and have a marginal therapeutic window at best. The fact that mefloquine is orally bioavailable and already approved for use in humans should facilitate its preclinical testing in combination with aminoglycosides to investigate the therapeutic potential of this approach. Mefloquine has been used for decades for malaria prophylaxis and treatment. Its side effects are well-documented and include abnormal dreams, insomnia, anxiety, depressed mood, nausea and dizziness [31]. Concerns have been raised about cases of depression and suicide associated with the use of mefloquine. However, a recent systematic review reported no suicides could be reliably attributed to mefloquine prophylaxis [31]. Combining a newer generation PTC readthrough aminoglycoside derivative such as NB124 with an approved enhancer like mefloquine has potential to generate sufficient functional readthrough *TP53* to achieve a therapeutic effect in cancer.

## Supporting information

S1 Fig Digitonin extraction. HDQ-P1 cell pellets were suspended in lysis buffer containing 0, 50, 100, 250, 500 or 1000 μg/ml digitonin. After centrifugation, the supernatants (cytosolic fraction) were collected and the remaining pellets were resuspended in NP-40 lysis buffer to extract the organellar fraction. The levels of cytosolic protein GAPDH and the organellar (lysosomal) protein TPP1 were determined in all fractions by automated capillary electrophoresis western analysis. The numbers show band intensity as % of the signal in samples in the first lane.
(TIF)

S2 Fig Effect of mefloquine on lysosomal acidification. HDQ-P1 cells were seeded at 1.5 x 10^4^ per well in 96-well plates. The next day, they were exposed to mefloquine at 0, 3, 10, and 30 μM for 24 h. HDQ-P1 cells exposed to the vacuolar ATPase inhibitor bafilomycin A1 (30 nM, LC Laboratories) were used as positive control for inhibition of lysosomal acidification. At the end of treatment, the cells were exposed to 150 nM LysoTracker Red (DND-99, Invitrogen) in fresh medium for 1 hour in a 37°C CO_2_ incubator. The wells were then rinsed twice with PBS and fixed with 3% paraformaldehyde, 1 μg/ml Hoechst 33323 in PBS for 30 min at room temperature, rinsed twice with PBS and stored overnight at 4°C. Imaging was carried out using a Cellomics ArrayScan VTI automated fluorescence microscope. Each bar is mean ± SD of four technical replicates.
(TIF)

S1 File Minimal data set of this study. (XLSX)
