# Supplementary figures and images for: The antimalarial drug mefloquine enhances TP53 premature termination codon readthrough by aminoglycoside G418
Source: PLoS One. 2019 May 23;14(5):e0216423. doi: 10.1371/journal.pone.0216423 (PMC6532957; doi:10.1371/journal.pone.0216423)

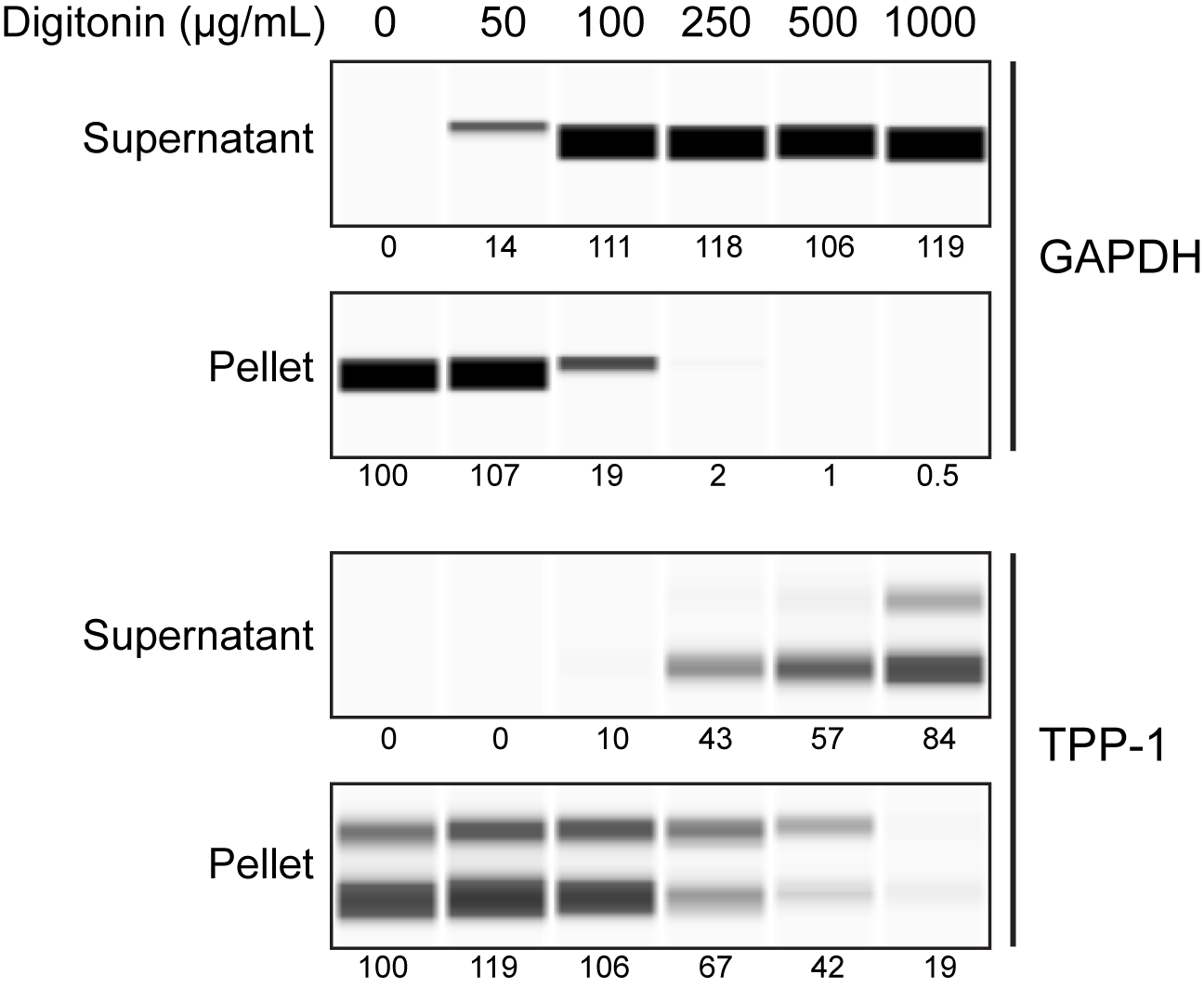

Supplement: S1 Fig — HDQ-P1 cell pellets were suspended in lysis buffer containing 0, 50, 100, 250, 500 or 1000 μg/ml digitonin. After centrifugation, the supernatants (cytosolic fraction) were collected and the remaining pellets were resuspended in NP-40 lysis buffer to extract the organellar fraction. The levels of cytosolic protein GAPDH and the organellar (lysosomal) protein TPP1 were determined in all fractions by automated capillary electrophoresis western analysis. The numbers show band intensity as % of the signal in samples in the first lane. (TIF) [file pone.0216423.s001.tif]

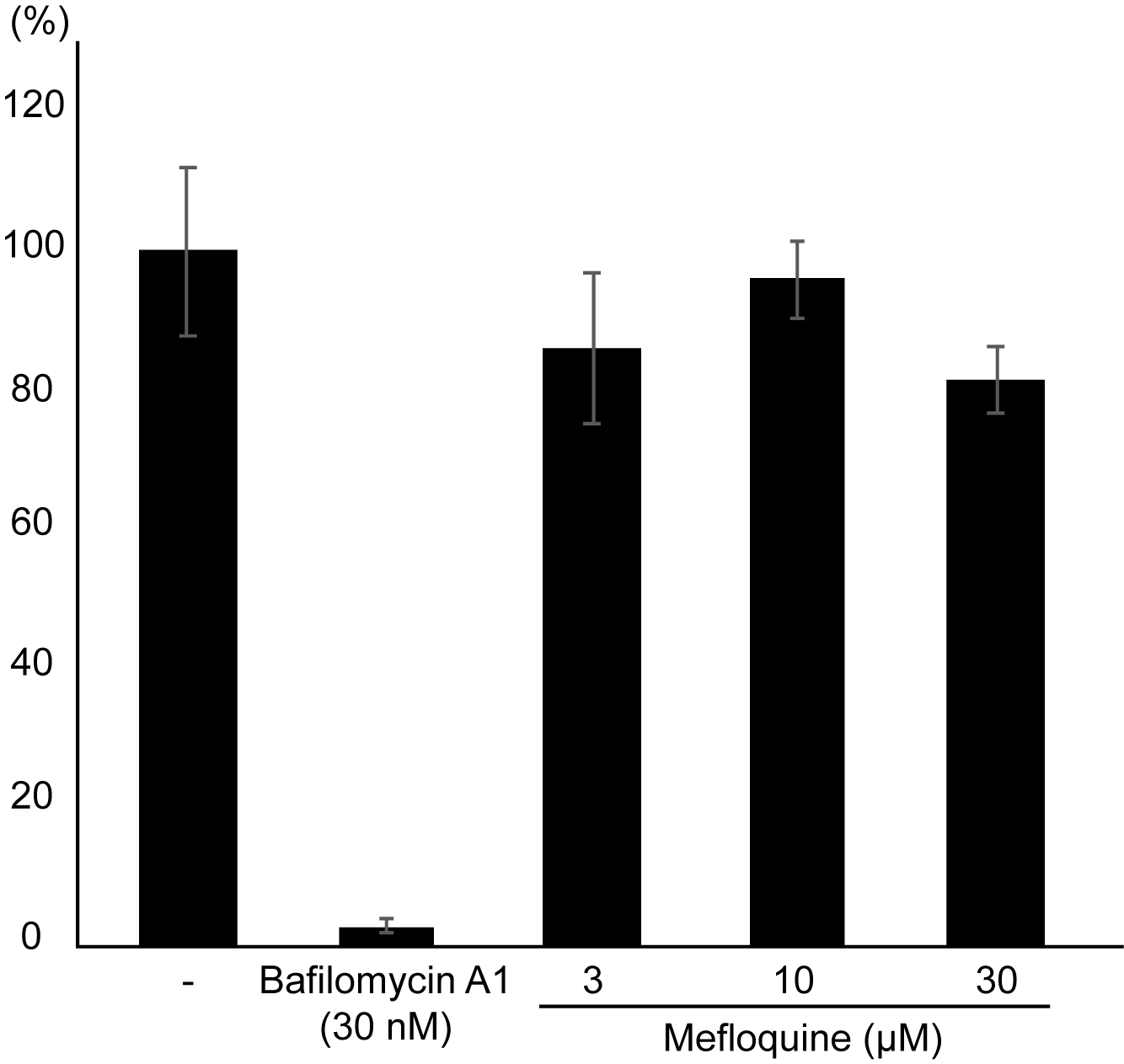

Supplement: S2 Fig — HDQ-P1 cells were seeded at 1.5 x 10^4^ per well in 96-well plates. The next day, they were exposed to mefloquine at 0, 3, 10, and 30 μM for 24 h. HDQ-P1 cells exposed to the vacuolar ATPase inhibitor bafilomycin A1 (30 nM, LC Laboratories) were used as positive control for inhibition of lysosomal acidification. At the end of treatment, the cells were exposed to 150 nM LysoTracker Red (DND-99, Invitrogen) in fresh medium for 1 hour in a 37°C CO2 incubator. The wells were then rinsed twice with PBS and fixed with 3% paraformaldehyde, 1 μg/ml Hoechst 33323 in PBS for 30 min at room temperature, rinsed twice with PBS and stored overnight at 4°C. Imaging was carried out using a Cellomics ArrayScan VTI automated fluorescence microscope. Each bar is mean ± SD of four technical replicates. (TIF) [file pone.0216423.s002.tif]
